# Quantitative anatomy of the ilium’s primary ossification center in the human fetus

**DOI:** 10.1007/s00276-018-2018-0

**Published:** 2018-04-19

**Authors:** Mariusz Baumgart, Marcin Wiśniewski, Magdalena Grzonkowska, Mateusz Badura, Maciej Biernacki, Zygmunt Siedlecki, Aleksandra Szpinda, Michał Szpinda, Katarzyna Pawlak-Osińska

**Affiliations:** 1 Department of Normal Anatomy, The Ludwik Rydygier Collegium Medicum in Bydgoszcz, The Nicolaus Copernicus University in Toruń, Łukasiewicza 1 Street, 85-821 Bydgoszcz, Poland; 2 Department of Neurosurgery, Neurotraumatology and Pediatric Neurosurgery, The Ludwik Rydygier Collegium Medicum in Bydgoszcz, The Nicolaus Copernicus University in Toruń, Bydgoszcz, Poland; 3 Department of Otolaryngology and Oncology, The Ludwik Rydygier Collegium Medicum in Bydgoszcz, The Nicolaus Copernicus University in Toruń, Bydgoszcz, Poland

**Keywords:** Ilium, Primary ossification center, Size, Growth dynamics, Regression analysis, Human fetuses

## Abstract

**Purpose:**

An understanding of the development of the ilium’s primary ossification center may be useful in both determining the fetal stage and maturity, and for detecting congenital disorders. This study was performed to quantitatively examine the ilium’s primary ossification center with respect to its linear, planar and volumetric parameters.

**Materials and methods:**

Using methods of CT, digital-image analysis and statistics, the size of the ilium’s primary ossification center in 42 spontaneously aborted human fetuses of crown–rump length (CRL) ranged from 130 to 265 mm (aged 18–30 weeks) was studied.

**Results:**

With no sex and laterality differences, the best fit growth dynamics for the ilium’s primary ossification center was modelled by the following functions: *y* = − 63.138 + 33.413 × ln(CRL) ± 1.609 for its vertical diameter, *y* = − 59.220 + 31.353 × ln(CRL) ± 1.736 for its transverse diameter, *y* = − 105.681 + 1.137 × CRL ± 16.035 for its projection surface area, and *y* = 478.588 + 4.035 × CRL ± 14.332 for its volume. The shape of the ilium’s primary ossification center did not change over the study period, because its transverse -to- vertical diameter ratio was stable at the level of 0.94 ± 0.07.

Conclusions

The size of the ilium’s primary ossification center displays neither sex nor laterality differences. The ilium’s primary ossification center grows logarithmically with respect to its vertical and transverse diameters, and linearly with respect to its projection surface area and volume. The shape of the ilium’s primary ossification center does not change throughout the examined period. The obtained quantitative data of the ilium’s primary ossification center is considered normative for respective prenatal weeks and may contribute to the prenatal ultrasound diagnostics of congenital defects.

## Introduction

The coxal bone displays a key role in the diagnostics of developmental defects of the lower limb and skeletal dysplasias. It is noteworthy that skeletodysplasias refer to a large and heterogeneous group of genetic defects, in which defective osseous or cartilaginous structures result from their inappropriate growth, development and differentiation [7, 14, 15, 18, 25]. Indeed, the overall incidence of skeletodysplasias is 1 case in 5000 live births that constitutes as many as 5% of children with congenital defects [10]. As a constituent of the hip joint, the ilium is a common subject of interest in numerous disciplines, such as anatomy, gynecology, obstetrics, sports medicine, manual therapy, biomechanics, anthropology and forensic medicine. The timing of ossification of three constituents of the coxal bone is well recognized [26, 35]. However, poor existing knowledge of the quantification of the primary ossification center of the ilium necessitates an investigation in this field. This is the first report in the professional literature to concentrate on the morphometric analysis of the ilium’s primary ossification center in the human fetus.

Therefore, the purposes of the present study were:

- to determine normative values for linear, planar and volumetric parameters of the ilium’s primary ossification center in human fetuses;
- to examine possible sex and laterality differences for all analyzed parameters;
- to describe the shape of the ilium’s primary ossification center over the examined period; and
- to compute growth dynamics for the analyzed parameters, expressed by best-matched regression models.

## Materials and methods

The study material comprised 42 human fetuses of both sexes (21 males and 21 females) of CRL ranged from 130 to 265 mm, and aged 18–30 weeks of gestation, originating from spontaneous miscarriages and preterm deliveries. The fetuses were collected before the year 2000 and still remain part of the fetal collection of our Department of Normal Anatomy. The experiment was approved by the Bioethics Committee of our University (KB 275/2011). The inclusion of the fetuses studied was based on the assessment of their external morphology and statistical cards with the course of pregnancy. Since on macroscopic examination neither internal nor external conspicuous morphological malformations were found, all included specimens were identified as normal. Of note, the fetuses did not display any developmental abnormalities of the musculoskeletal system. The fetal ages were determined on CRL [20] and the known date of the beginning of the last maternal menstrual period. Furthermore, the fetuses studied could not suffer from growth retardation, as the correlation between the gestational age based on CRL and that calculated by the last menstruation reached the value *R* = 0.97 (*p* < 0.001). Table 1 lists the characteristics of the study group, including CRL, number and sex of the fetuses.

**Table 1:** CRL, number and sex of the fetuses studied

CRL ranges (mm)	Crown–rump length (CRL) (mm)				Number of fetuses	Sex	
	Mean	SD	Min	Max		♂	♀
130–140	133.3	5.80	130.0	140.0	3	1	2
146–154	150.00	3.03	146.0	154.0	6	2	4
159–160	159.67	0.58	159.0	160.0	3	2	1
171–178	174.67	3.51	171.0	178.0	3	2	1
186	186.00	0.00	186.0	186.0	2	0	2
195–197	196.33	1.15	195.0	197.0	3	1	2
204–213	208.67	3.81	204.0	213.0	9	5	4
214	214.00	–	214.0	214.0	1	0	1
225–233	229.00	5.70	225.0	233.0	2	1	1
236–241	239.25	2.36	236.0	241.0	4	4	0
249–250	249.50	0.70	249.0	250.0	2	0	2
253	253.00	–	253.0	253.0	1	0	1
263–265	263.67	1.15	263.0	265.0	3	3	0
Total					42	21	21

**Fig. 1:**
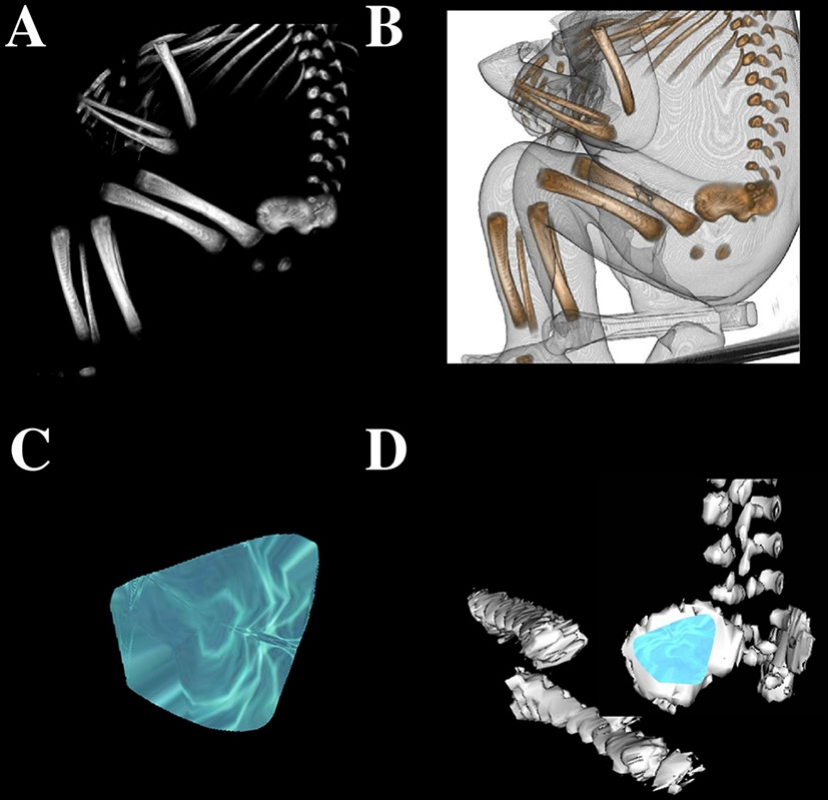
A female human fetus aged 23 weeks in the sagittal projection (**a**), its skeletal reconstruction (**b**), its volumetric reconstruction of the ilium’s primary ossification center (**c**), 3D reconstruction of the left ilium and its primary ossification center (**d**) using Osirix 3.9

**Table 2:** Vertical and transverse diameters, projection surface area and volume of the right ilium’s primary ossification center

CRL ranges (mm)	Number of fetuses	Primary ossification center of the right ilium							
		Vertical diameter (mm)		Transverse diameter (mm)		Projection surface area (mm^2^)		Volume (mm^3^)	
		Mean	SD	Mean	SD	Mean	SD	Mean	SD
130–140	3	8.66	0.20	8.31	0.23	58.93	6.22	119.00	2.14
146–154	6	9.04	0.33	8.77	0.18	65.22	1.85	122.83	0.73
159–160	3	10.10	0.74	9.50	0.66	72.15	1.20	134.19	12.46
171–178	3	12.44	0.29	11.97	0.77	106.49	7.62	222.05	52.27
186	2	13.10	0.08	12.90	0.19	96.17	8.06	256.50	8.74
195–197	3	13.56	0.23	13.27	0.59	116.33	4.59	320.51	49.53
204–213	9	13.72	0.17	12.85	1.41	120.04	10.41	335.25	50.68
214	1	14.08	–	11.90	–	116.00	–	340.63	–
225–233	2	14.80	0.09	13.98	0.08	138.70	7.95	426.23	58.38
236–241	4	16.17	0.28	15.34	0.71	150.91	9.41	487.11	63.21
249–250	2	16.89	0.14	15.74	1.36	178.88	2.69	507.24	9.01
253	1	17.30	–	16.60	–	182.70	–	580.75	–
263–265	3	18.01	0.75	18.31	1.38	206.41	20.19	590.12	54.71

**Table 3:** Vertical and transverse diameters, projection surface area and volume of the left ilium’s primary ossification center

CRL ranges (mm)	Number of fetuses	Primary ossification center of the left ilium							
		vertical diameter (mm)		transverse diameter (mm)		projection surface area (mm^2^)		volume (mm^3^)	
		mean	SD	mean	SD	mean	SD	mean	SD
130–140	3	8.64	0.16	8.56	0.12	58.47	5.53	118.88	0.70
146–154	6	8.99	0.36	8.54	0.26	64.60	2.71	122.35	3.43
159–160	3	10.02	0.99	9.02	0.01	69.10	0.28	138.81	10.16
171–178	3	12.37	0.34	11.72	1.52	105.66	12.83	229.51	42.79
186	2	13.20	0.14	12.93	0.28	98.97	7.48	238.40	10.67
195–197	3	13.55	0.87	11.81	1.82	114.23	12.30	337.30	66.27
204–213	9	13.93	0.26	11.94	0.86	118.89	11.32	340.74	54.89
214	1	14.03	–	12.13	–	118.20	–	342.00	–
225–233	2	14.60	0.22	13.79	0.79	130.98	13.75	438.45	58.47
236–241	4	16.39	0.37	14.29	1.02	155.04	19.04	488.01	63.04
249–250	2	16.76	0.12	15.68	0.91	187.90	0.00	524.93	1.84
253	1	17.50	–	16.98	–	199.70	–	524.93	1.84
263–265	3	18.03	0.88	17.30	0.92	212.00	21.33	594.22	53.17

Using a Siemens-Biograph 128 mCT camera (Siemens Healthcare GmbH, Erlangen, Germany) situated at Department of Positron Emission Tomography and Molecular Imaging (Oncology Center, Collegium Medicum of the Nicolaus Copernicus University, Bydgoszcz, Poland), the fetuses were scanned at a step of 0.4 mm, recorded in DICOM formats (Fig. 1), and subsequently subjected to morphometric analysis with the use of the Osirix 3.9 software. It should be emphasized that Osirix 3.9 allows for precise numerical analysis of any linear, planar and three-dimensional reconstructions of objects studied. The gray scale of achieved CT pictures expressed in Hounsfield units (HU) ranged from − 275 to − 134 for a minimum, and from + 1165 to + 1558 for a maximum. Thus, the window width (WW) altered from 1.404 to 1.692, and the window level (WL) varied from + 463 to + 712. The specifics of the imaging protocol were as follows: mAs – 60, kV – 80, pitch – 0.35, FoV – 180, rot. time – 0.5 s., while the specifics of CT data were: slice thickness – 0.4 mm, image increment – 0.6 mm, and kernel – B45 f-medium. Of note, both WW and WL optimize the appearance of CT images by determining the contrast and brightness levels assigned to the CT image data. WW directly refers to the maximal number of shades of grey to be displayed on a CT monitor, and expressed by the range of HU. WL is referred to as the midpoint of the range of the CT numbers displayed (window center) (Table 2).

Despite the cartilaginous developmental stage, precise contours of the ilium’s primary ossification center were already evidently visible [9, 17], and so a morphometric analysis regarding its linear, planar and spatial parameters was feasible. To precisely visualize and measure the ilium’s primary ossification center, the resulting fetal scans must have been rotated with relation to the three reference axes: vertical (cranial-caudal), horizontal and sagittal, to finally reach a reference position. It is noteworthy that in such a required position, the vertical, horizontal and sagittal axes always traversed the very center of the ilium’s primary ossification center, and were set at right angle to each other. Due to these maintained landmarks, the consistency in measurements was absolute. Additionally, such a position of these three axes made the ilium’s primary ossification center set accurately in the sagittal projection (Table 3).

**Fig. 2:**
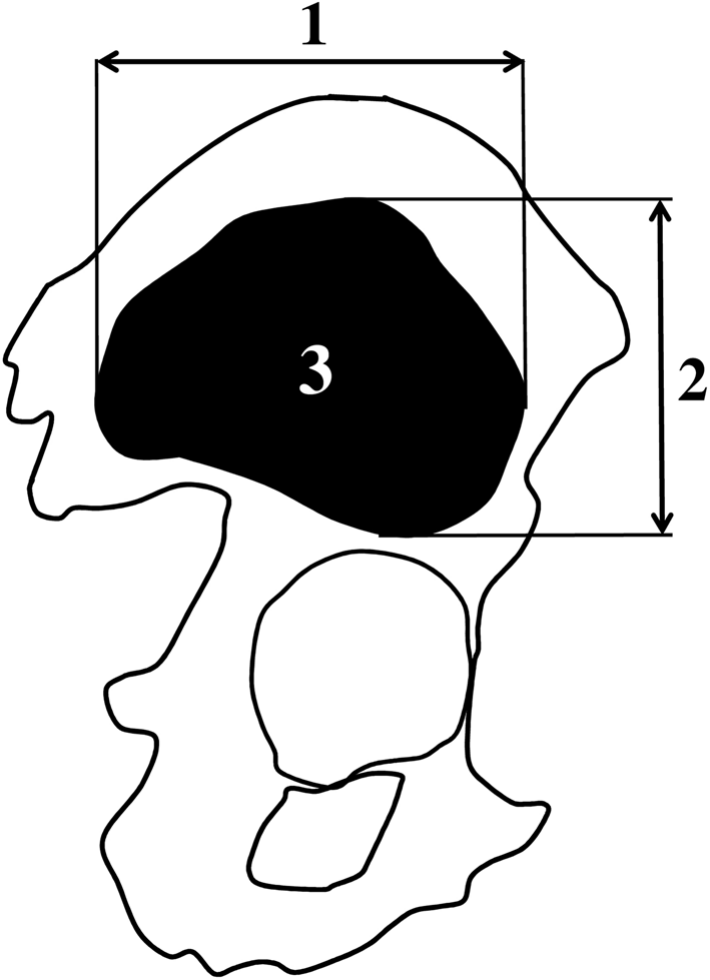
Measurement scheme of the ilium’s primary ossification center in the sagittal plane. 1—Vertical diameter, 2—transverse diameter, 3—projection surface area

Measurements of the ilium’s primary ossification center were conducted in a specific sequence (Fig. 2). In each fetus, the quantitative evaluation of the ilium’s primary ossification center was bilaterally carried out, concerning its four parameters:

1. vertical diameter, based on the determined distance between its upper and lower borderlines in the sagittal plane (Fig. 2),
2. transverse diameter, based on the determined distance between its anterior and posterior borderlines in the sagittal plane (Fig. 2),
3. projection surface area, based on the outlined area occupied by the ossification center in the sagittal plane on the lateral view (Fig. 2), and
4. volume, calculated using advanced diagnostic imaging tools for 3D reconstruction, taking into account the absorption of radiation by bony tissue (Fig. 1c).

Besides, to examine the shape of the ilium’s primary ossification center we calculated its transverse -to- vertical diameter ratio.

All measurements were performed by one researcher (M.B). Each measurement was performed three times under the same conditions but at different times, and averaged. The results obtained were statistically analyzed. Distribution of variables was checked using the Shapiro–Wilk (*W*) test, while homogeneity of variance was checked using Fisher’s test. The results were expressed as arithmetic means with standard deviations (SD). To compare the means, the Student *t* test for independent variables and one-way analysis of variance were used. Tukey’s test was used for post hoc analysis. If no similarity of variance occurred, the non-parametric Kruskal–Wallis test was used. The characterization of developmental dynamics of the examined parameters was based on linear and curvilinear regression analysis. The match between the numerical data and computed regression curves was evaluated based on the coefficient of determination (*R*^2^).

## Results

The statistical analysis revealed neither significant sex nor bilateral differences, which allowed us to compute only one growth curve for each analyzed parameter. On both the right and left sides, the growth dynamics of the vertical and sagittal diameters of the ilium’s primary ossification centers followed natural logarithmic functions.

**Fig. 3:**
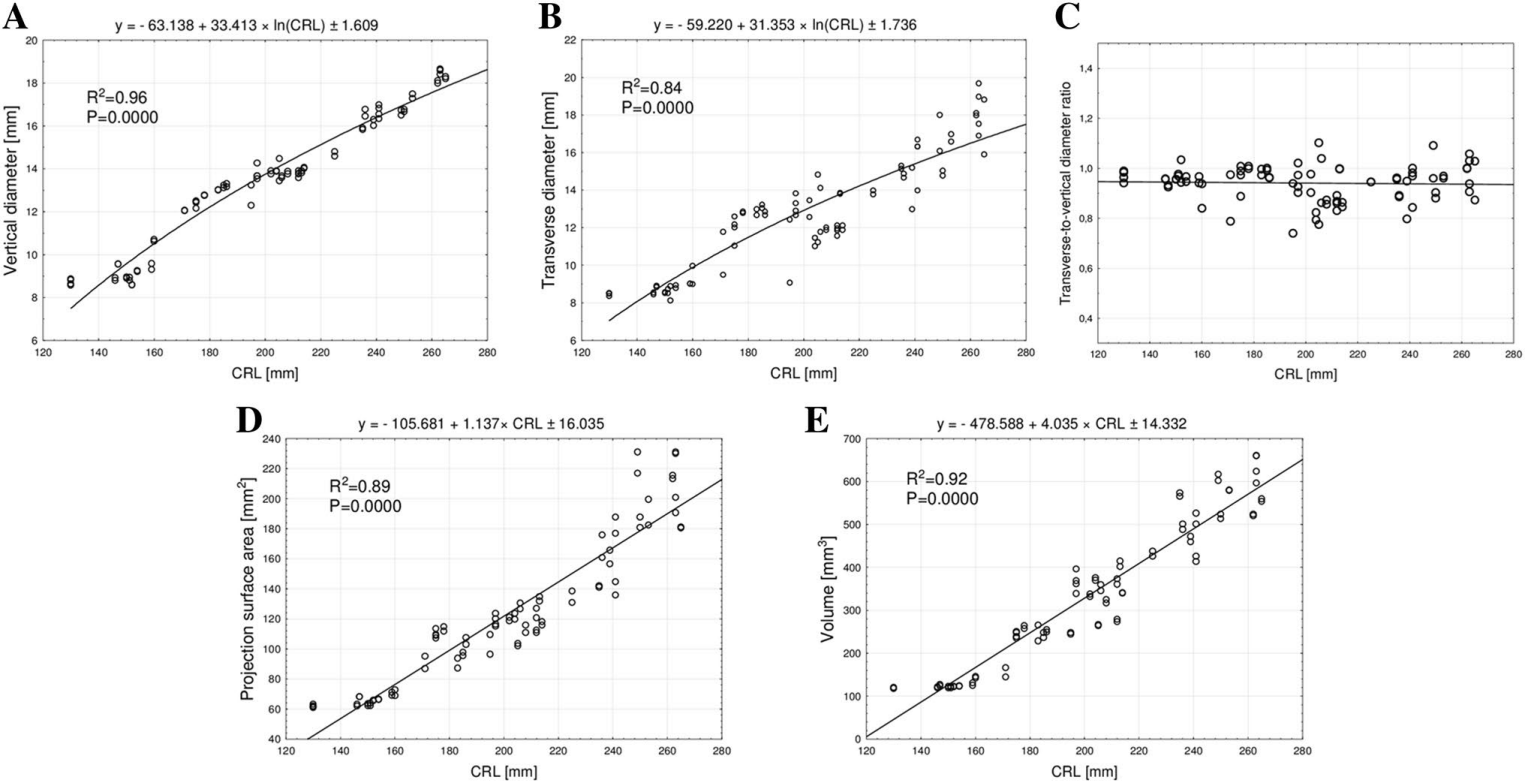
Regression lines for vertical diameter (**a**), transverse diameter (**b**), transverse – to vertical diameter ratio (**c**), projection surface area (**d**), and volume (**e**) of the ilium’s primary ossification center

The mean vertical diameter of the ilium’s primary ossification center in fetuses of CRL ranged from 130 to 265 mm grew from 8.66 ± 0.20 to 18.01 ± 0.75 mm on the right, and from 8.64 ± 0.16 to 18.03 ± 0.88 mm on the left, following the natural logarithmic function *y* = – 63.138 + 33.413 × ln(CRL) ± 1.609 (*R*^2^ = 0.96) —(Fig. 3a).

The mean transverse diameter of the ilium’s primary ossification center in fetuses of CRL ranged from 130 to 265 mm grew from 8.31 ± 0.23 to 18.31 ± 1.38 mm on the right, and from 8.56 ± 0.12 to 17.30 ± 0.92 mm on the left, in accordance with the natural logarithmic function: *y* = – 59.220 + 31.353 × ln(CRL) ± 1.736 (*R*^2^ = 0.84)—(Fig. 3b).

Throughout the examined period the shape of the ilium’s primary ossification center did not change. This was expressed by its transverse -to- vertical diameter ratio, at the constant level of 0.94 ± 0.07 (Fig. 3c).

The mean projection surface area of the ilium’s primary ossification center between CRL ranged from 130 to 265 mm increased from 58.93 ± 6.22 to 206.41 ± 20.19 mm^2^ on the right, and from 58.53 ± 5.53 to 212.00 ± 21.33 mm^2^ on the left, following the linear function: *y* = – 105.681 + 1.137 × CRL ± 16.035 (*R*^2^ = 0.89)—(Fig. 3d).

The mean volume of the ilium’s primary ossification center in the fetuses studied grew from 119.00 ± 2.14 to 590.12 ± 54.71 mm^3^ on the right, and from 118.88 ± 0.70 to 594.22 ± 53.17 mm^3^ on the left, in accordance with the linear function: *y* = – 478.588 + 4.035 × CRL ± 14.332 (*R*^2^ = 0.92)—(Fig. 3e).

## Discussion

The method of choice for evaluating the fetal anatomy is routine ultrasound [1]. Identifying developmental defects e.g. skeletodysplasias in utero fetuses is mostly based on reduced dimensions of long bones in relation to gestational age, abnormal morphological features or bone mineralization, and the presence of fractures. However, the effectiveness of ultrasonic examination ranges from 40 to 60%, and so the use of ultrasound alone does not suffice to comprehensively diagnose some abnormalities, such as a narrow thorax. When any skeletal dysplasia is suspected, diagnostic imaging using radiography [21], ultrasonography [23], computed tomography [34], and magnetic resonance imaging [1, 11, 30] is essential. Victoria et al. [32] and Cassart et al. [8] demonstrated a higher diagnostic precision using 3D CT compared to 2D ultrasound in skeletal dysplasias. A currently limiting factor for CT examinations is a lack of numerical data describing the fetal skeletal system at consecutive weeks of gestation in comparison with ultrasound examinations. However, a great advantage of the CT technique is the possibility to observe the examined structure in any plane, and at any time without sacrificing image details after the examination [34]. Compared to 2D X-ray, computed tomography eliminates the overlap of anatomical structures and allows for an easy distinction between different body tissues. Nowadays, MRI becomes an increasingly powerful adjuvant for investigating the fetal anatomy, both in utero and post-mortem. Even without fetal sedation, due to faster MRI sequences done during suspension of maternal breathing, the quality of MRI images has relatively been increased [1]. The fetal anatomy at MRI is extremely indispensable in the 2nd and 3rd trimesters when ultrasound imaging is either equivocal or just limited by a lack of an adequate acoustic window, e.g. low amniotic fluid volume (oligohydramnios) and breech presentation [11]. Because of the recent advancement of fetal surgery, the use of fetal MRI mostly refers to congenital diseases of the central nervous system, skeletal system and thoraco-abdominal viscera [1]. Lately developed cine-MRI techniques provide a novel insight into movements of an entire fetus in the three-dimensional uterine environment during pregnancy [30].

The process of ossification of the ilium commences at week 9 of gestation [14, 15] in the perichondrium adjacent to a nucleus of disintegrating cartilage cells, just above the greater sciatic notch at the superior edge of the acetabulum [12, 13, 22, 31, 35], and progresses cephalad towards the iliac crest [15, 31]. As stated by Laurenson [22] in his histological study performed on seven human fetuses of CRL ranging from 38 to 100 mm, growth of the primary ilium’s ossification center follows an ossification pattern similar to that in the humeral shaft. In the 38 mm fetus, the presumptive ossification center was intensely basophilic and contained osteoblasts and bone formation. In the 50 mm fetus, an osseous shell spread cephalad over either surface of the ilium’s ala, without invading the subjacent cartilage. However, in the 58 mm fetus, pores in the ilium’s ala emerged, through which osteoblasts and vessels invaded the disintegrated cartilage with the formation of the primary medullary cavity. The ischium ossifies much later—at month 4 of gestation, while the pubis ossifies between months 4 and 5 of the prenatal life. During that time, outlines of the greater sciatic notch, as well as the anterior superior and anterior inferior iliac spines are already discernible. The growing primary ossification centers of the coxal bone contribute to the formation of the acetabulum, the three parts of which remain separated by a Y-shaped cartilage layer until puberty [14, 15, 31]. The development of the ilium has certain characteristic features. First, from week 15 of gestation until birth, the lateral part of the ilium is always 2–3 times thicker than its medial part, which may be caused by the activity of the gluteal musculature. Second, from month 6 of gestation, a small amount of cartilage-like tissue, very sensitive to pathogenic factors, appears within the upper posterior part of the acetabulum, which may cause a pathological development of the acetabulum under adverse conditions [14, 15, 18]. Thirdly, the process of ossification of the ilium resembles the ossification of long bones that have two epiphyseal cartilages and one cartilaginous diaphysis [14, 22].

Furthermore, secondary ossification centers of the acetabulum are formed at the age of 10 years in girls, and at 12–13 years in boys. Fusion of the three primary ossification centers occurs between 4 and 6 years, while in the acetabulum—between 12 and 18 years of age. Between the age of 13 and 15, the ossification centers that ultimately model the shape of the coxal bone are formed, i.e. those for the anterior inferior iliac spine, ischial spine, ischial tuberosity and iliac crest, the last of which remains unfused for a relatively long time—until the age of 21–25 years. The ossification center for the pubic tubercle and the subpubic angle is formed between the age of 18 and 20 years [7, 16, 18].

The ilium plays a critical role in transferring loads while seating, standing and walking. Movements accompanying these actions exert structural changes in the bone architecture from the embryonic period to adult age [31]. Recent work from the Nowlan Group [19, 25, 29, 30] has found fetal movements to be important for joint shape of the human hip, potentially resulting in developmental dysplasia of the hip (DDH). Apart from being a significant indicator of general fetal health, fetal movements are critical for musculoskeletal development, and so decreased fetal movements of neuromuscular origin may produce diverse skeletal abnormalities like hypo-mineralized bones, joint fusions, deformed joint shapes and craniofacial malformations [19, 25, 30]. Significantly, abnormal or reduced fetal movements, fetal breech position, particularly extended breech with the hips flexed and knees extended, joint laxity and restricted nulliparous uterine cavities lead to increased risk of DDH [19] and osteoarthritis in the later life [29, 30]. Various changes to the high forces exerted by the fetal iliopsoas muscle during kicking probably have a critical effect on the biomechanical stimuli experienced by the hip joint [30], likely because the resulting muscle forces generate stress and strain within the fetal skeleton, and so stimulate the developing skeletal tissues [29]. Additionally, Ward and Pitsillides [33] suggested that the left hip has a higher risk of DDH than the right one due to the common position of the fetal left leg beside the mother’s spine, which limits hip abduction. As stated by Giorgi et al. [19], a full range of symmetric fetal movements tends to minimize the natural tendency of decreasing stability at the developing hip between gestational week 11 and birth, by helping to maintain both the acetabular depth and femoral head sphericity. Contrariwise, reduced or absent fetal movements may lead to decreased femoral head roundness and its acetabular coverage, while abnormal asymmetric fetal movements may result in a deformed hip joint shape, with a shallow asymmetric acetabulum and a somewhat malformed femoral head. Of note, fetal kick force increased significantly over time, from 29 to 47 N between weeks 20 and 30, before decreasing significantly to 17 N at week 35 [29].

In this study, we demonstrated that in terms of quantity the ilium’s primary ossification center did not demonstrate any sex or bilateral differences. It is interesting to note that Mokrane et al. [24] found no sex differences in the fetal ilium, as well. Similarly, previous studies to examine the influence of sex on the size of different ossification centers revealed no sex differences with relation to the humerus [32], femur [3], clavicle [2], as well as C1, C2 [4, 5], C4 [6], T6 [28] and L3 [27] vertebrae in human fetuses. It should be emphasized that the present study is the first to quantitatively evaluate the size and growth dynamics of the ilium’s primary ossification center as a function of fetal CRL. The linear parameters of the ilium’s primary ossification center increased logarithmically, following the functions *y* = – 63.138 + 33.413 × ln(CRL) ± 1.609 for its vertical diameter, and *y* = – 59.220 + 31.353 × ln(CRL) ± 1.736 for its transverse diameter. Furthermore, both its projection surface area and volume increased in a commensurate fashion: *y* = – 105.681 + 1.137 × CRL ± 16.035, and *y* = – 478.588 + 4.035 × CRL ± 14.332, respectively. Unfortunately, a lack of numerical data concerning the ilium’s primary ossification center in the medical literature limits a more detailed discussion on this topic. Its noteworthy that the shape of the ilium’s primary ossification center was virtually unchanged throughout the examined period. This was supported by its transverse-to-vertical diameter ratio, the value of which persisted at the level of 0.94 ± 0.07.

The obtained morphometric data regarding the ilium’s primary ossification center may be useful in the diagnostics of skeletal dysplasias that are often characterized by a disrupted or limited fetal growth. The commonest congenital defects of the coxal bone include DDH, which results in a dislocation of the femur due to deformation of the acetabulum and femoral head [31]. Hypoplasia of the coxal bone accompanied by an enlargement of lateral parts of the iliac alae and a decrease in the acetabular angle is typical of Down syndrome. Of note, this anomaly can already be detected in in utero fetuses. In 80% of individuals suffering from Down syndrome, an enlargement of the iliac alae, shallowing of the acetabular dome and an increase in the curvature of the femur can be noted [23]. Similar signs are usually observed in achondroplasia and lethal dysplasias. An achondroplastic pelvis is often described as “tombstone-shaped pelvis”, “Mickey Mouse ear pelvis” or “champagne glass pelvis”.

## Conclusions

1. The morphometric characteristics of the ilium’s primary ossification center display neither sex nor laterality differences.
2. The ilium’s primary ossification center grows logarithmically in its vertical and transverse diameters, and linearly in its projection surface area and volume.
3. The shape of the ilium’s primary ossification center is virtually unchanged throughout the examined period.
4. The obtained quantitative data of the ilium’s primary ossification center is considered normative for respective prenatal weeks and may contribute to the prenatal ultrasound diagnostics of congenital defects.
